# “Hot Hand” on Strike: Bowling Data Indicates Correlation to Recent Past Results, Not Causality

**DOI:** 10.1371/journal.pone.0030112

**Published:** 2012-01-12

**Authors:** Gur Yaari, Gil David

**Affiliations:** 1 Department of Pathology, Yale School of Medicine, New Haven, Connecticut, United States of America; 2 Department of Mathematics, Program in Applied Mathematics, Yale University, New Haven, Connecticut, United States of America; University of Maribor, Slovenia

## Abstract

Recently, the “hot hand” phenomenon regained interest due to the availability and accessibility of large scale data sets from the world of sports. In support of common wisdom and in contrast to the original conclusions of the seminal paper about this phenomenon by Gilovich, Vallone and Tversky in 1985, solid evidences were supplied in favor of the existence of this phenomenon in different kinds of data. This came after almost three decades of ongoing debates whether the “hot hand” phenomenon in sport is real or just a mis-perception of human subjects of completely random patterns present in reality. However, although this phenomenon was shown to exist in different sports data including basketball free throws and bowling strike rates, a somehow deeper question remained unanswered: are these non random patterns results of causal, short term, feedback mechanisms or simply time fluctuations of athletes performance. In this paper, we analyze large amounts of data from the Professional Bowling Association(PBA). We studied the results of the top 100 players in terms of the number of available records (summed into more than 450,000 frames). By using permutation approach and dividing the analysis into different aggregation levels we were able to supply evidence for the existence of the “hot hand” phenomenon in the data, in agreement with previous studies. Moreover, by using this approach, we were able to demonstrate that there are, indeed, significant fluctuations from game to game for the same player but there is no clustering of successes (strikes) and failures (non strikes) within each game. Thus we were lead to the conclusion that bowling results show correlation to recent past results but they are not influenced by them in a causal manner.

## Introduction

Nowadays, more and more data sets are available in a digital format allowing for researchers to address questions which were practically impossible before. Sports is a great example of a research area that benefit tremendously from current information era (eg. [1]–[10]). The “hot hand” phenomenon has gained huge interest in the last 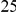 years since Gilovich, Vallone and Tversky published their benchmark paper about it [11]. They argued that what people tend to perceive as “hot hand” or “streakiness” in sports is due to their wishful thinking that the world is not as random and not due to the actual data observed (see also the reviews of [12], [13] and the website [14]). The opposite belief is the gambler's fallacy in which people tend to think that totally random sequences (like casino machines) balance themselves for past results: for example, if a coin was flipped 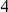 times resulting in 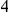 tails, most gamblers will give more chances for a head in the next flip even if they are completely positive that the coin is fair. These purely psychological theories have a lot of relevancy in other fields such as economics, and could be thought of as conceptual descendants of the ground breaking works of Daniel Kahneman and Amos Tversky [15], [16], which paved the way to the prospect theory [17] on which Daniel Kahneman had received the Nobel prize in 2002. Sports is a wonderful setup to test these ideas since it involves both athletes and observers. The athletes aspect adds an interesting layer to this phenomenon by allowing the possibility that athletes can potentially change their success rates based on recent past results. This is an important point, which people tend to mix together with the “hot hand” phenomenon: the appearance of correlation to recent past results (the “hot hand” definition) does not necessarily imply that there is a *causal* connection between current results and the one to be obtained next (for the difference between correlation and causality see for example [18], [19] or somehow more popular explanations in [20], [21]). This is to say that even if there is a strong evidence in support of the presence of the “hot hand” phenomenon in a specific data set (which lately has been shown in sevatter, is aneral data sets [3], [5]–[7], [9], [10], [22] ), it does not mean that the results are a consequence of an underlying (psychological/physiological) feedback mechanism (causality)! The latter is an extremely interesting question on its own in psychology in general and sports psychology in particular. The consequences of knowing if such feedback mechanism exists or not could potentially help in choosing the right strategy for succeeding in a sports competition and also in more generic tasks.

The original “hot hand” paper [11] studied this phenomenon inside the world of sports; in particular, in basketball, where the researchers performed a set of experiments where they asked the players and observers about their perceptions of the results and compared them to what they could detect in the data. Since they did not find any significant positive correlation between successive shots, they concluded that the “hot hand” is a phenomenon that exists only in the observers and players minds. One of the data sets analyzed in the original paper was composed of all free throws taken by the Boston Celtics during 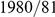 season of the NBA. This data set was reanalyzed later by Wardrop [23], who demonstrated how the typical observer could actually be right: although he could not find deviations from the null hypothesis of repeated independent trials in the individual level, he argued that what the observers store in their minds is one big data set which includes all players. This data set aggregates the results of better and worse shooters leading to the apparent appearance of a “hot hand” (see [7], [23]). Since, in the individual level, Wardrop did not find traces of this phenomenon and concluded by justifying the original conclusions in [11]. In another study [22], Wardrop showed that the statistical tests used in [11] weren't sensitive enough to detect small deviations from the null hypothesis. This was demonstrated further by Korb and Stillwell [24]. In his study, Wardrop was able to use other tests and detected the “hot hand” in a controlled setting: he analyzed 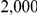 shots taken by one player (Katie Voigt, who took 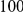 throws each day for 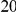 days). Apart from this example involving one individual only, the review of Bar Eli et al. [12] showed only a handful of examples that could potentially be interpreted as supporting the existence of the hot hand phenomenon (in particular [5], [22], [25]). Since this review, it was shown that in basketball [6], [7] the “hot hand” phenomenon does exist and streakiness was also found in [3], [6], [9]. However, in a recent paper we hypothesized [7] that the patterns observed in the data are due to “better” and “worse” periods (good and bad days) rather than to a psychological/physiological mechanism that causes the performance of the player to change due to previous results. The only apparent solid evidence of causality or causal dependency between consecutive trials was shown in [5]. They have analyzed bowling data and argued that the data is neither stationary nor independent. However, we found that the test used by them (which then was extended by [8]) to detect non independence wasn't satisfactory and instead could have detected non stationarity in the probability of success rather than independence (in the sense of *causal* dependency between the results, see supporting information S1 for details).

Similar to [5], [8], [26], hereby we analyze large data from PBA (we studied 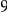 seasons as oppose to one by [5]) and show that “hot hand” traces are indeed evident in the data at the individual level. However, this phenomenon is present only when data is aggregated between different games. Within each game there is no evidence for a deviation from the null hypothesis of repeated independent Bernoulli trials with constant probability of success. Hence, we reach the conclusion that there is no *causal* connection between the result of one frame and the next one.

## Results

### Data

A bowling game is composed of 10 frames. In each frame the player has 1–2 attempts to roll a ball from the start line of a lane aiming to knock down pins, located 60 feet down the lane. Each frame's starting setup is built of ten pins arranged in an equilateral triangle shape with one of its perpendicular bisectors pointing to the bowler along the center of the lane. A strike is called when a bowler knocks all ten pins down on the first attempt. If not all ten pins are knocked down, the fallen pins are removed from the lane and the bowler has a second attempt to knock the remaining pins down. A spare is called in case all the remaining pins are knocked down on the second trial. If some pins remain up after the second roll, the frame is called open. In case the 10th frame is a spare or a strike, the bowler is given a bonus roll for a spare and two rolls for a strike.

### Scoring scheme

Each frame contributes to the bowler's score the number of pins knocked down in that frame. In addition, if there is a spare or a strike on that frame, the score of the next roll (in case of spare) or two rolls (in case of strike) is added to the bowler's score. A perfect game consists of 12 strikes (ten frames plus two bonus rolls), which results in the highest possible score: 300.

### The dataset

The PBA website [27] contains data of bowling games that were played between 1959 and 2011. For the 2002–2011 seasons, it presents frame by frame scores of games from selected rounds (usually the more advanced rounds in each tournament). We downloaded the available data from 2002 to 2011 and extracted 47,653 games that had corresponding frame by frame scores. These games were associated to 374 individual players (men, women and seniors), where the minimum number of frame by frame games per player was 3 and the maximum was 1501. We ordered the players according to the number of frame by frame games per player and selected the top 100 players. This decision was made in order to improve our statistical power and have less noise due to insufficient statistics for some individuals. The filtered dataset included 41,966 frame by frame games, where the minimum number of frame by frame games per player was 82 and the maximum was 1501. This list overlaps significantly with other ranking schemes such as overall earnings or mean score.

### Pre-processing of the dataset

For the current analysis (as in [5], [8]), we transformed each game into a binary sequence of 10–12 time points: 10 for the first 10 frames (1 for strike and 0 otherwise) and an extra 1–2 time points in cases of bonus attempts. Since the result (0,1) is not possible for the pair associated with time points (11,12), we trimmed the results of time point 12 (if existed) from each game. This trimming process avoids biases, noise and complications. At the end of this pre-processing process, our dataset included 459,423 frames that were used for the analysis step.

### Analysis

**Figure 1:**
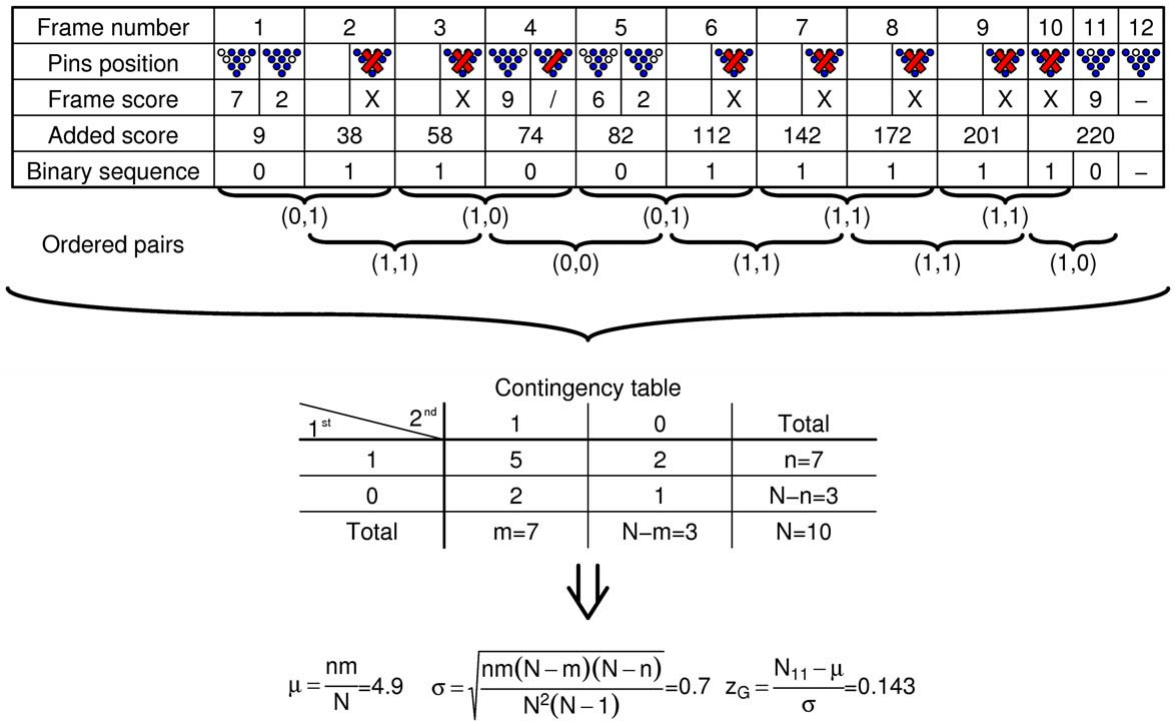
Example of calculating 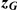 for one game. The top four rows of the top table show a typical scoring box of a bowling game. The fifth row is our way of transforming this information into a binary sequence of success/failure (strike/non strike) for each frame. This series is broken into (ten in this case) ordered pairs of consecutive results, which in turn populate the bottom contingency table. From this table one can use the hypergeometric distribution to obtain an expectation (mean) and variance for the upper left cell 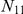. The final step is to calculate a 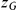 value for this table based on the expectations and the observed value.

For each individual player *j*, we have considered separately four different levels of aggregation: games, tournaments, seasons and career (G/T/S/C). Then, for each level of aggregation we calculated 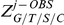 as follows:

1. From each binary sequence (a game) a collection of 9–10 ordered binary pairs were extracted from all consecutive pairs of indexes ((1,2),(2,3)…(10,11)). 9 in case there were 10 time points in the game and otherwise 10 (recall that the 12th result was omitted from the presented analysis).
2. For each member of the aggregation level *i* (e.g the *i*th season), all ordered pairs belonging to *i* were combined to form one contingency table 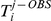.

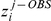
 was computed from 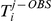 (see also figure 1) as: denoting the number of 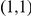
's as 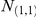, the number of 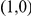
's as 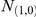 etc., we can use the hypergeometric distribution to obtain:
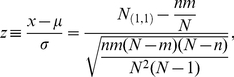
(1)where 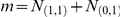, 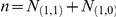 and 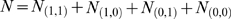.

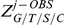
 was computed based on the the number of existing members in each level 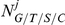 (number of games/tournaments/seasons/career player 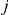 had results for) as
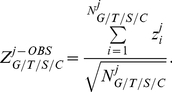
(2)

In principal, we could have taken a different route and instead of using the “z-statistics”, we could have calculated an exact Fisher/Bernard test for each table. This route is dubious due to the fact that even “exact” tests are problematic when the numbers are small [28], [29]; moreover, we are interested in combining many P values from different tables into one “q value” (a P value of a collection of P values); the standard method of doing it [30], will be further affected by the discrete nature of the data. Another statistical test sometimes used for this kind of problems is the Cochran-Mantel-Haenszel test [31], however it assumes homogeneity of the different games [32], an assumption we wanted to avoid; it also suffers from discreteness effects as well. Due to this “hostile” statistical environment, we chose a different permutation approach, which takes advantage of the z-statistics. We tested this method on simulated data (see below) and obtained very good results.

In general, z-statistics is used in cases where 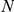 is large enough (and for Binomial distributions, the probability of success, 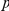, is not too extreme) to obtain a P value using the cumulative normal distribution. Then, one can obtain a P value for each table (one player and one item in the aggregated level (e.g. game)) and a “q value” for this player using the same cumulative normal distribution but instead of plugging in it the 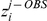 from equation 1, use the 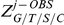 from equation 2. However, in the current data 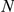 is 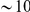 for games and 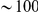 for tournaments. Therefore a simple use of the z-statistics will not help to confront the intrinsic difficulties discrete variables bring with them. After we observed that 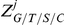 is not centered around zero in the data, we have checked the distribution of 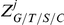 for simulated data (see supporting information S1); we measured a significant bias in the mean of 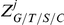, which diminishes as aggregation level elevates and 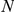 increases. The permutation approach we have adopted, has proven to be very useful and sensitive for our purposes (see below the validation of this approach).

**Figure 2:**
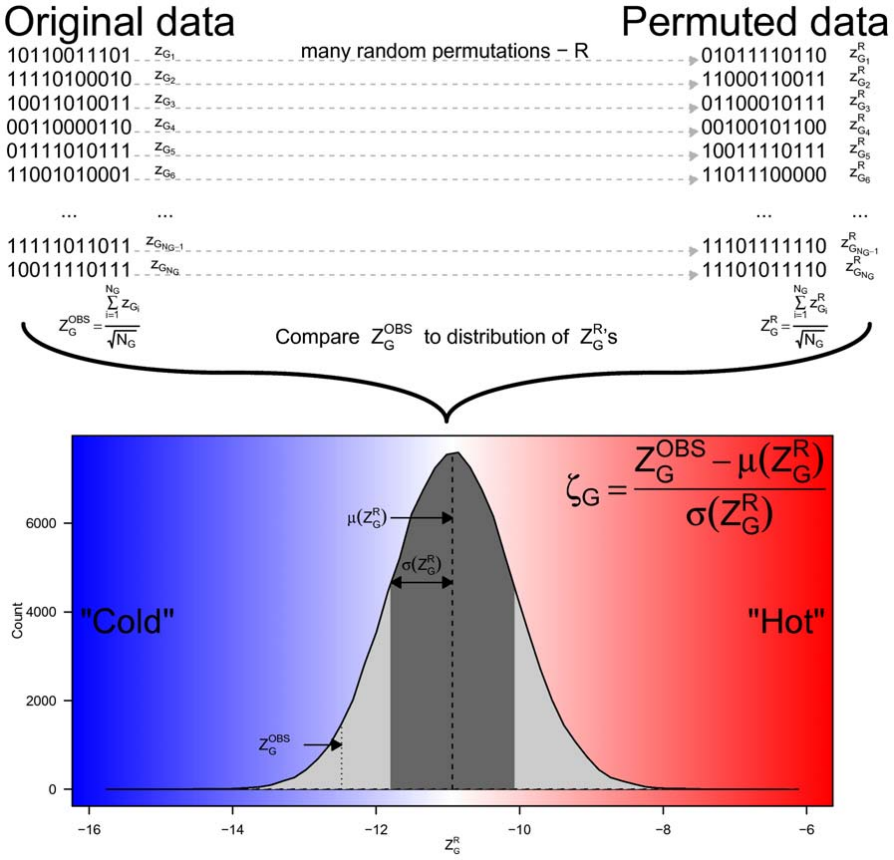
Example of calculating 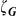 for one player. On the upper left we show how for binary sequence 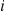 (a game) we calculate 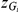 (see figure 1) which are averaged to obtain 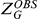. On the upper right we show how for each binary sequence we make a random permutations of it (R) and then calculate the corresponding 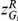. For each realization (R) we calculate 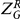, save this value and repeat these steps many times (100,000). On the bottom plot we show a typical distribution of 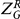 (in this case it is for player number 11534, Walter Ray Williams Jr who happen to show negative correlation to previous roll); since this distribution is very close to a normal distribution we can take advantage of it and measure the deviation of the observed value (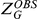) from the expected one (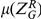) in units of the standard deviation (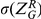). If the observed value falls into the right of the mean (red area), it means the player has a “Hot hand” tendency while if the observed value falls in to the left of the mean (blue area), it mean the player has “Cold hand” (“anti hot hand”). The resulting 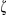 reflects the statistical significance of this observation.

In short, what we did, is for each player ( *j* ) and each aggregation level we calculated 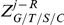, which is an equivalent of 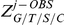, but for a randomly permuted data. The permutation was done within each member of the aggregation level; e.g. for season *i*, the binary results from all games belonging to this season (excluding the 12th result of each game if exists) were re-shuffled amongst the different games of that season to obtain a permuted set of results. After obtaining a large number (100,000 in our case) of random permutations of the data for each player and each aggregation level, a distribution of 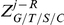 is produced and 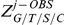 can be compared to this distribution to achieve a statistical answer to how “hot”/“cold” this player is. Since the resulting distributions of 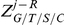 had a normal shape (see figure 2 and supporting information S1 for examples), we could use the 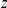 statistics once again to asses statistical significance of the result. In order to decrease ambiguity we named the resulting value for each player and each aggregation level 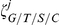, which was defined as:
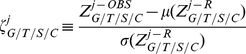
(3)The reader is referred to figures 1 and 2 in which a schematic flow of the process is presented: figure 1 demonstrate how data from a real bowling game is transformed into a binary sequence and then to a set of ordered pairs, which populate a contingency table from which a 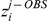 value is obtained. Figure 2 shows how 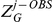 (the level of aggregation referred to in this figure is games) is compared to the distribution of 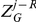 to obtain a 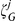 value for this player.

The last step in the analysis was to estimate whether the collection of 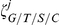 for the different players agrees with random results: this was done using a simple 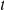 test.

### Validating the permutation approach using stochastic simulations

In order to gain insight on the sensitivity of the permutation approach, we have considered an imaginary, typical, player who played in 5 seasons, 20 tournaments in each season, each of which had 10 games. The base probability of success of this player was set to p = 0.6 (which is close to the mean value of the top 100 players, 0.579) and several scenarios were considered: The base probability for each game (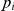) was drawn from a uniform distribution between 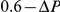 and 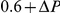. The result of the 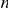
th frame (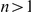) in game 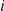 was drawn from a Bernoulli distribution with parameter 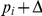 if the result of the 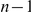 frame was a success and 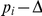 otherwise.

**Figure 3:**
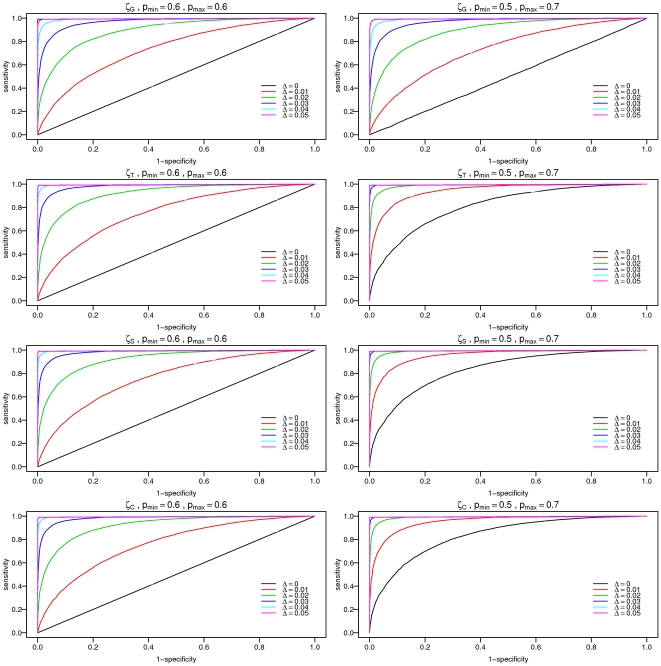
Method validation with stochastic simulations. ROC curves are calculated for different values of 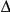 and 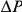 following the stochastic simulations described in the text. The left column refers to 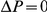 while the right column refers to 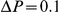. The different rows correspond to different aggregation levels of the test (games, tournaments, seasons and career: G/T/S/C). One sees how the presented method starts to show impressive power for very small values of 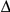. However, aggregation levels of T/S/C detect streakiness for 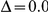 while in aggregation level G, it is not detected as designed.

With this setup we could separate the effects of nonstationarity (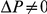) and independence/causality (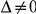). We ran 10,000 realizations for each parameter value considered and recorded the resulting 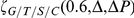. The distributions of 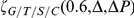 can be compared to the distribution of 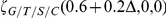 to check for sensitivity and specificity. In the supporting information S1 we plot these distributions for 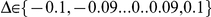 and 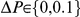. In figure 3, we plot the resulting ROC curves from these distributions. One sees how all of the tests detect 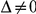 with good power even for small 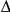
's: for 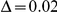, a threshold of 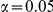 and 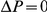, one can get a power of 0.53 for the 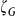 based test and 0.63 for the tests based on 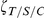. In the cases where 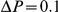, the corresponding powers are 0.53 for the 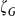 based test (same as for the previous case), 0.94 for 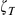 and 0.96 for the tests based on 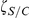. However, one sees that the tests based on 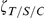 all show very high false positive rates - the black curves in figure 3, which are supposed to be the identity lines, are very far off and give (false) power of 0.33 for a threshold of 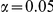 and 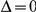 for 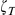 and 0.37 for the tests based on 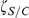 (for the right column in figure 3, where 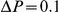). A generalization of the presented permutation approach could potentially be useful for other kinds of time series (e.g financial) in which one is interested in the timescales where “good” periods and “bad” periods alternate.

### The “hot hand” results from nonstationarity, not causality between consecutive trials

**Figure 4:**
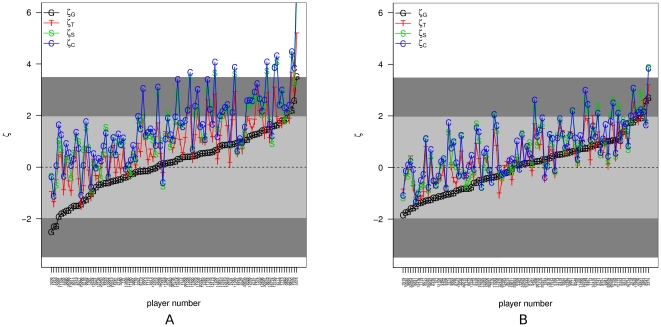
Significance of deviation from NULL hypothesis for different aggregation levels. The top 50 players (in terms of number of available games in the last 9 seasons) are ordered according their 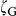. In this plot, 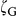, 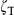, 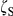 and 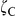 are presented. The shaded areas reflect the 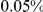 alpha threshold and the same value corrected for multiple tests (100 here). Panel a is for all games exist in the data, panel b is round 16 and above only.

The main result of the current paper is presented in figure 4, where the four different aggregation levels (games/tournaments/seasons/career) results are presented for the top 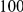 players of the PBA. The names of the players can be extracted from the supporting information S1. One can see that the collection of results for 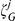 is centered around zero in a nice way. The mean of 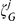 is 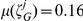 and the standard deviation is 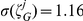; t test for this distribution results in a non significant P value of 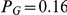. However, the distribution of the other aggregation levels are biased towards positive values (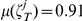, 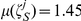, 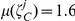) monotonously with aggregation level resulting in very significant P values (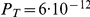, 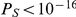 and 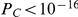 for the tournaments/seasons/career respectively from a t test).

This implies two major observations that present in the data:

1. The result of previous roll *does not* affect the result of the current roll;
2. The probability of success is not constant between games, even within tournaments.

These observations result from the fact that for the games aggregation level, the results agree with random order (high P values), which means no clustering of success and failure within each game, and do not agree with random order (very low P values) for higher aggregation levels.

Indeed, it has been shown in bowling that games results seem to depend on previous games and are not independent from each other [26]. One possible explanation for the nonstationarity in the probability of success within tournaments is that some games are more important than others leading to a decrease in concentration of the players leading them to perform worse in the less important games. To further study this effect, we repeated the entire analysis on a subset of the data: only top 16 games of each tournament (only the top 16 players of each tournament are still counted) and up. Though we have less data points in this subset, leading to a reduced statistical power, we still obtain similar qualitative picture of “neutral hand” within games and “hot hand” between games due to nonstationarity (see figure 4B). This indicates that the fluctuations in the probability of success are not due to the importance of the game but rather to other possible factors influencing players performance.

In summary, we showed that players performance in bowling deviates significantly from a simple repeated independent Bernoulli trials with constant probability of success. Nonetheless, this is not due to a simple psychological feedback loop, in which the result of current frame is influenced by the result of previous frame, but rather to more complex factors. These, currently undetermined, factors lead to the appearance of “hot hand” when data is aggregated across several games. Within each game, however, we could not rule out the null hypothesis of repeated independent trials with constant probability of success. This is to say that if indeed these complex factors, which determine players performance, are connected to psychological impact of past performance, this is not in a time scale which is shorter than a game.

### Gauging effect

**Figure 5:**
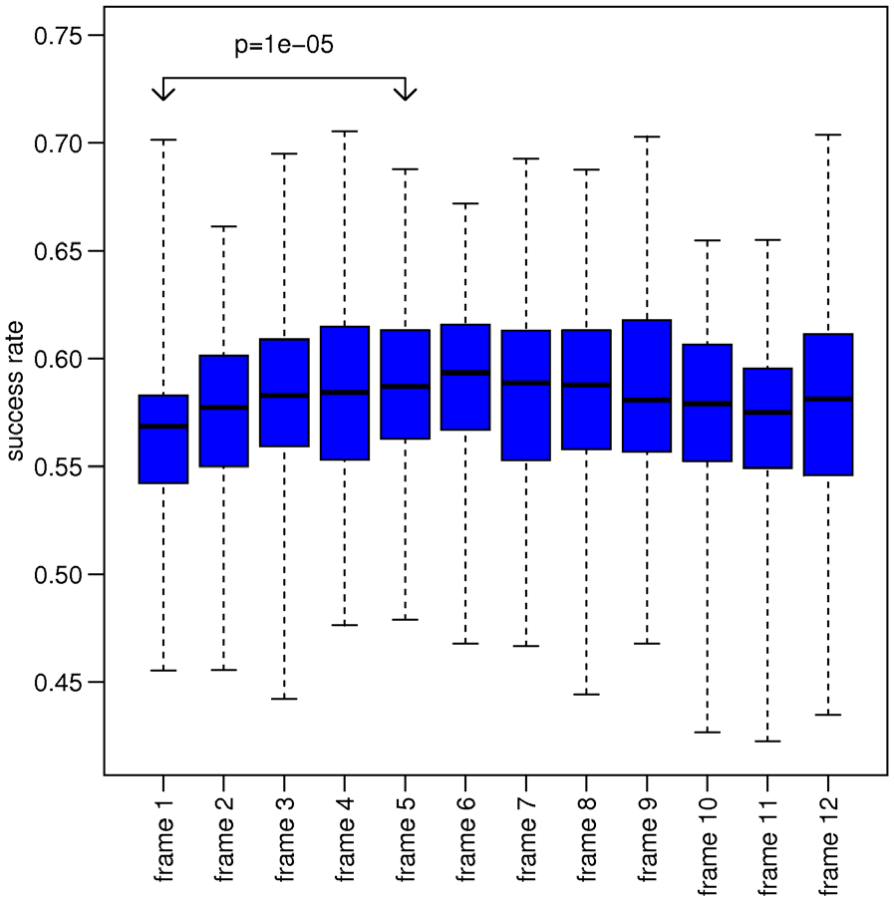
Success rates by frame for the top 100 players. Box plot of the mean probability of success of each player for the different frames in a bowling game. The increase in the mean (black solid line) success rate of all players between frame 1 and frame 3 is evident. A paired Mann-Whitney test (two sided) between frame 1 and frame 5 yields a very low P value of 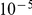 (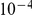 between frame 1 and 3). The dashed line spans the mean success rate of all of the players while the blue rectangle spans the middle 50% quantiles.

Yet another pattern we were able to observe in the data is the fact that the first and second frames of a game, typically have lower success rates than the following ones. Although noisier, one can see in figure 4 how the mean success rate of the top 100 players increases in 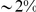 from the first frame to the 3rd onwards. Statistical significance (e.g. for the difference between frame 1 and 5 as shown in figure 5) was calculated using a paired Mann-Whitney test (two sided). This effect resembles the finding reported in [7], [23] about the fact that in basketball, the second free throw has higher success rate than the first one in a sequence of two consecutive free throws. One could think of a possible explanation for this phenomenon as some kind of “gauging effect” in which the player is able to fine tune the direction of the ball following a recent trial due to muscle short term memory.

### Gaining prediction power

**Figure 6:**
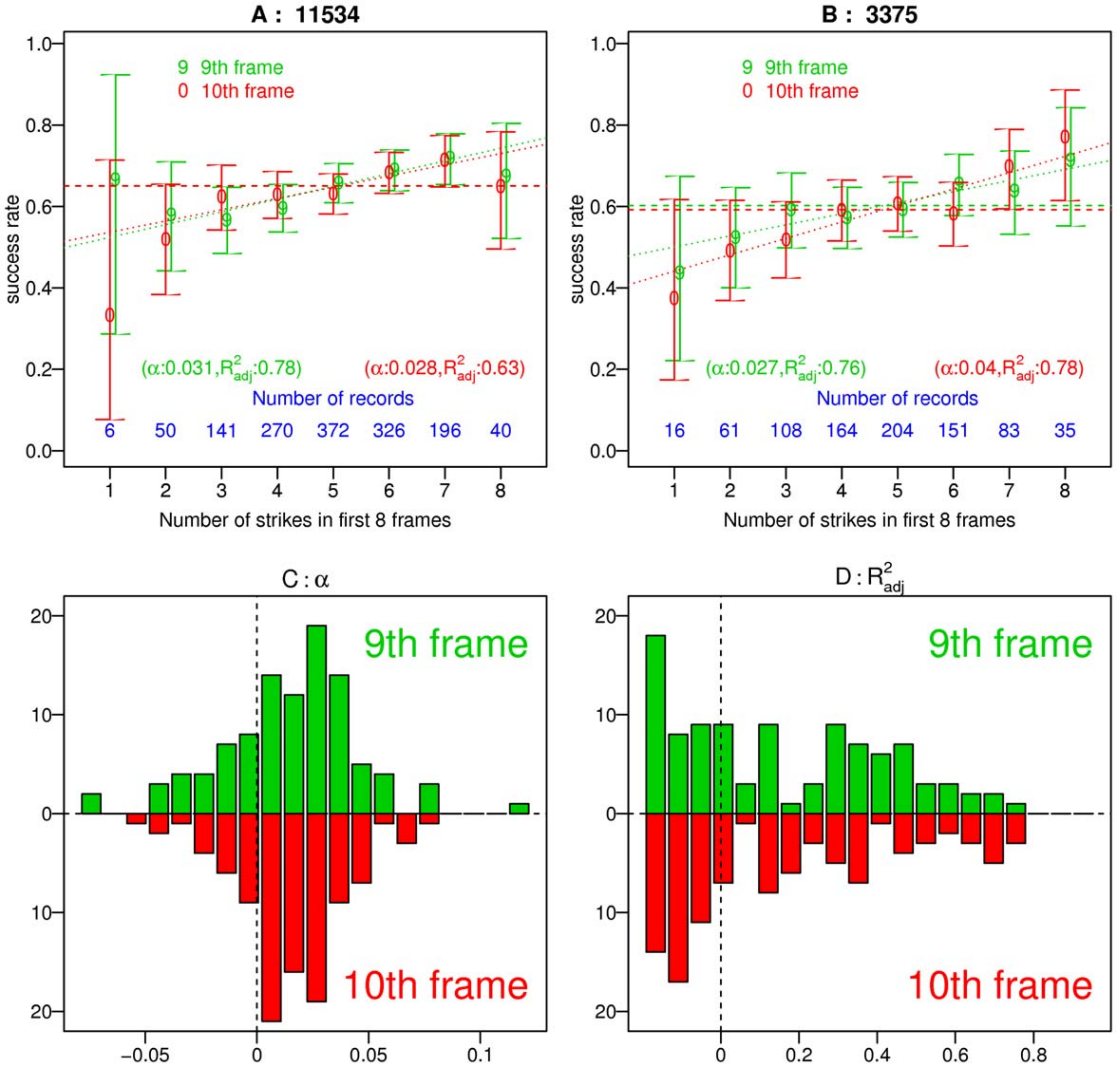
Gaining prediction power. Panels A–B: The conditional probability for a strike in the 9th (green) and 10th (red) frames given the number of strikes in the first 8 frames is plotted. The 95% confidence intervals shown are calculated using bayesian framework with Jeffreys prior (see [33]). Data is shown for two individuals with the most identifiable patterns. The numbers at the title of each panel represents the player's code according to the supporting information S1. Dashed lines represent the base probability of success for each player in the corresponding frame and dotted lines show the linear fits results. Panels C and D show the distribution of slopes (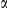) and 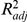 obtained from the fits of all players respectively.

We showed that the “hot hand” is present when data is aggregated across games and argued that this is due to “better and worse” games. An interesting question is whether we can tell if a specific game is “good” or “bad” based on its beginning. To address this question we measured for each player the probability of rolling a strike in the 9th (and 10th) frame given that there were N strikes in the first 8 frames (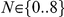). As expected from our prior observation (individuals have “good” game or a “bad” games), indeed we found an increasing trend in the probability of success with the number of strikes rolled in the first 8 frames. As in the case with the “hot hand” analysis, this trend is not true for all individuals and is quite noisy as data is limited. Figure 6 summarizes our findings with this regards: for each individual, we made a linear fit between the probability of success in the 9th (10th) frame and the number of strikes rolled in the first 8 frames. Weights were given based on the 95% confidence intervals, calculated using a Bayesian framework with Jeffreys prior [33]. Panels A and B in that figure show the most significant trends observed for two individuals. In panel C, one can see that indeed most of the fits yielded positive trends; p values for such deviations calculated with a simple t test yield very small values (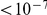). However, since data is limited for each individual the quality of fit is not always as good as for the two cases shown. In panel D, one can see that the distribution of 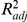 [34] is centered around small values most likely due to small numbers of observations which yields large confidence intervals. In general, if data is sufficient one can use such method to improve the prediction power for the last frames of each game. Practically speaking, in any case the “binary” prediction for these frames will be a strike as for the players analyzed here, the probability of rolling a strike is (almost always) larger than 50% regardless of the first 8 frames. Nonetheless, if one uses some kind of gambling strategy (e.g. the Kelly strategy [35]), this result might help in deciding regard the amount of money to bet on (given that such gambles do exist and legal).

## Discussion

We showed that in bowling data the appearance of a “hot hand” is evident in the individual level. By using a permutation approach, which was proven to be very sensitive in this kind of data, we were able to prove that the underlying mechanism behind the appearance of this phenomenon is time fluctuations in the probability of success rather than a psychological/physiological feedback loops. The fact that we can show that human activities do not follow a constant probability of success may not surprise many of us. However, this was not the consensus in the scientific community in the past three decades since the work of Gilovich Vallone and Tversky. Indeed, one could argue whether human beings can detect such a relatively delicate statistical pattern from occasional typically short and incomplete observation they observe. Without entering this debate, the current work may suggest that people tend to detect “hot hand” trends as an adaptive strategy [36], [37], which could allow for identifying trends in human (or other) activities as generally they do not follow a random independent process with constant probability of success.

The lack of causality between current roll and previous one may open the door for new experiments, which will try to identify the local causes that alter the performance of the player. Although in this research we study bowling data, the question of what influence individual performance (or concentration) is fundamental and of high importance. What we have showed here is that the alternation between “good” periods and “bad” periods is not due to a simple feedback mechanism between current results and past results. The question of what actually determine the quality of performance of human subjects remains open and most likely is task depended.

## Supporting Information

Supporting Information S1
**Appendix and supporting figures and table.** In the appendix we show that the test used by [5], [8] to detect non independence might detect non stationarity in the probability of success rather than independence (in the sense of *causal* dependency between the results). The figures show examples of distributions of 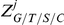 for simulated data and the table links players numbers and their real names.(PDF)

## References

[1] Heuer A, Mller C, Rubner O (2010). Soccer: Is scoring goals a predictable poissonian process?. EPL (Europhysics Letters).

[2] Radicchi F Who is the best player ever? a complex network analysis of the history of professional tennis.. PLoS ONE.

[3] Sire C, Redner S (2009). Understanding baseball team standings and streaks.. The European Phys- ical Journal B.

[4] Duch J, Waitzman JS, Amaral LAN (2010). Quantifying the performance of individual players in a team activity.. PLoS ONE.

[5] Dorsey-Palmateer R, Smith G (2004). Bowlers' hot hands.. The American Statistician.

[6] Arkes J (2010). Revisiting the hot hand theory with free throw data in a multivariate framework.. Journal of Quantitative Analysis in Sports.

[7] Yaari G, Eisenmann S (2011). The hot (invisible?) hand: Can time sequence patterns of suc- cess/failure in sports be modeled as repeated random independent trials?. PLoS ONE.

[8] Martin DEK (2006). Hot-hand effects in sports and a recursive method of computing probabilities for streaks.. Comput Oper Res.

[9] McCotter T (2010). Hitting streaks don't obey your rules: Evidence that hitting streaks aren't just By-Products of random variations.. Chance.

[10] Raab M, Gula B, Gigerenzer G (2011). The hot hand exists in volleyball and is used for allocation decisions.. Journal of Experimental Psychology:Applied.

[11] Gilovich T, Vallone R, Tversky A (1985). The hot hand in basketball: On the misperception of random sequences.. Cognitive Psychology.

[12] Bar-Eli M, Avugos S, Raab M (2006). Twenty years of hot hand research: Review and critique.. Psychology of Sport and Exercise.

[13] Alter AL, Oppenheimer DM (2006). From a fixation on sports to an exploration of mechanism: The past, present, and future of hot hand research.. Thinking & Reasoning.

[14] Reifman DA (2009). The hot hand in sports.. http://thehothandblogspotcom/.

[15] Tversky A, Kahneman D (1971). Belief in the law of small numbers.. Psychological Bulletin.

[16] Tversky A, Kahneman D (1974). Judgment under uncertainty: Heuristics and biases.. Science.

[17] Tversky A, Kahneman D (1979). Prospect theory: An analysis of decision under risk.. Econometrica.

[18] Holland PW (1986). Statistics and causal inference.. Journal of the American Statistical Association.

[19] Pearl J (2009). Causality: Models, Reasoning and Inference.

[20] Watson G (2004). http://256.com/gray/thoughts/2004/20040511.html.

[21] Munroe R (2009). http://xkcd.com/552/.

[22] Wardrop RL (1999). Statistical tests for the hot-hand in basketball in a controlled setting.. http://www.stat.wisc.edu/~wardrop/papers/tr1007.pdf.

[23] Wardrop RL (1995). Simpson's paradox and the hot hand in basketball.. The American Statistician.

[24] Korb KB, Stillwell M The story of the hot hand: Powerful myth or powerless critique?.

[25] Albright SC (1993). A statistical analysis of hitting streaks in baseball.. Journal of the American Statistical Association.

[26] J A (2002). Ladder tournaments and underdogs: lessons from professional bowling.. Journal of Economic Behavior and Organization.

[27] (2011). http://pba.com.

[28] Yates F (1984). Test of significance for 2×2 contingency tables.. Journal of the Royal Statistical Society Series A (General).

[29] Agresti A, Coull BA (1998). Approximate is better than “Exact” for interval estimation of binomial proportions.. The American Statistician.

[30] Mosteller F, Fisher RA (1948). Questions and answers.. The American Statistician.

[31] Mantel N, Haenszel W (1959). Statistical aspects of the analysis of data from retrospective studies of disease.. J Natl Cancer Inst.

[32] Agresti A (2007). An introduction to categorical data analysis.

[33] Agresti A, Min Y (2005). Frequentist performance of bayesian confidence intervals for comparing proportions in 2×2 contingency tables.. Biometrics.

[34] Theil H (1961). Economic forecasts and policy. Contributions to economic analysis.

[35] Kelly JJ (1956). A new interpretation of information rate.. Information Theory, IRE Transactions on.

[36] Burns BD (2001).

[37] Wilke A, Barrett HC (2009). The hot hand phenomenon as a cognitive adaptation to clumped resources.. Evolution and Human Behavior.

