# Supplementary material for: “Hot Hand” on Strike: Bowling Data Indicates Correlation to Recent Past Results, Not Causality
Source: PLoS One. 2012 Jan 12;7(1):e30112. doi: 10.1371/journal.pone.0030112 (PMC3257263; doi:10.1371/journal.pone.0030112)
Supplement: Supporting Information S1 — Appendix and supporting figures and table. In the appendix we show that the test used by [5], [8] to detect non independence might detect non stationarity in the probability of success rather than independence (in the sense of causal dependency between the results). The figures show examples of distributions of for simulated data and the table links players numbers and their real names. (PDF) [file pone.0030112.s001.pdf]

SUPPORTING INFORMATION:

"Hot hand" on strike: Bowling data indicate correlation to recent past results, not causality.

Gur Yaari[1,*], Gil David[2]
1 Department of Pathology, Yale School of Medicine, New Haven, CT 06511, USA
2 Department of Mathematics, Program in Applied Mathematics, Yale University, New Haven, CT 06510, USA
∗ E-mail: gur.yaari@yale.edu

1 Test for independence or rather nonstationary?

Dorsey-Palmateer and Smith [1] and Martin [2] considered a situation where a strike in Bowling is a success and a non-strike is a failure. They calculated the probability of a success given that the previous j trials were all successful and compared it to the probability of a success given that the previous j trials were all failures. They have obtained from their data systematic deviations between these two values and concluded that this is a sign for dependency between the current trial and the previous one(s). We show that their conclusion does not imply *causal dependency* between current result and previous one as this test detects also nonstationary probability of success (with repeated *independent* trials). In order to show it, let us hypothesis a non stationary Bernoulli independent trials with the following parameters: the system switches between two modes, 1 and 2. A fraction F of the time the system is in mode 1 and a fraction $1-F$ the system is in mode 2. The switchings are done either in time lags drawn from a Poisson distribution without any dependency of the current or past results or in fixed times. When the system is in mode 1 the probability of success is p_1 while when it is in mode 2, the probability of success is p_2. One can calculate the overall success rate given that the last j trials were all failures to be

$$P(1|\overbrace{0,0....,0}^{j \text{ times}}) = \frac{Fp_1(1-p_1)^j + (1-F)p_2(1-p_2)^j}{F(1-p_1)^j + (1-F)(1-p_2)^j} \tag{1}$$

vs. the opposite scenario where all the last j trials were a success

$$P(1|\overbrace{1,1....,1}^{j \text{ times}}) = \frac{Fp_1^{j+1} + (1-F)p_2^{j+1}}{Fp_1^j + (1-F)p_2^j} \tag{2}$$

. These expressions are different (when $p_1 \neq p_2$) and depend on j what prove the claim.

Figures

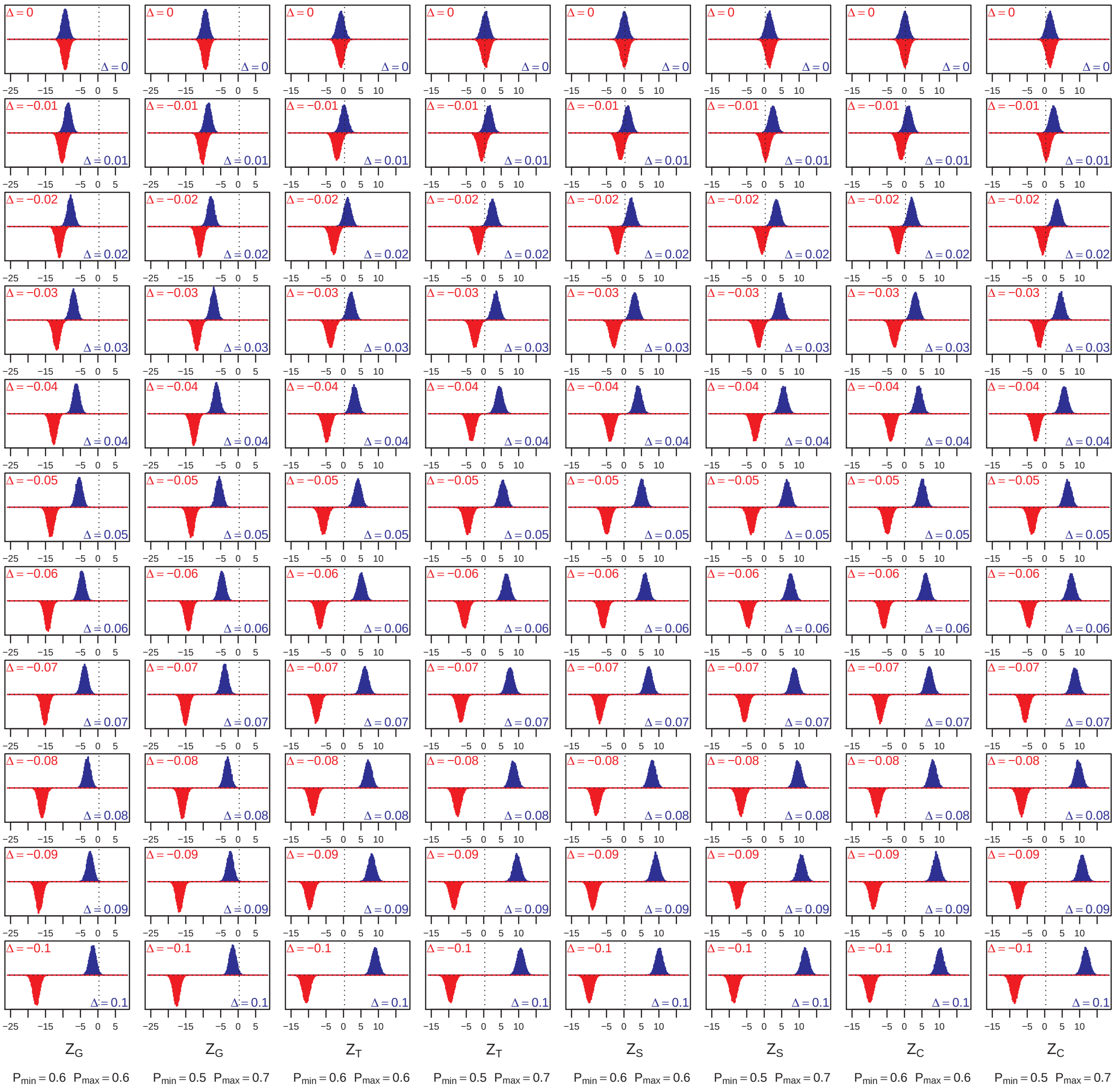

Figure 1. Method validation with simulations
Distributions of Z for different aggregation levels (games, tournaments, seasons and career: G/T/S/C) and different values of Δ and ΔP. Different rows correspond to different values of Δ. Blue bars refer to positive Δ's while red refer to negative Δ's. The different columns correspond to differetn aggregation levels and different values of ΔP's as indicated in the legends.

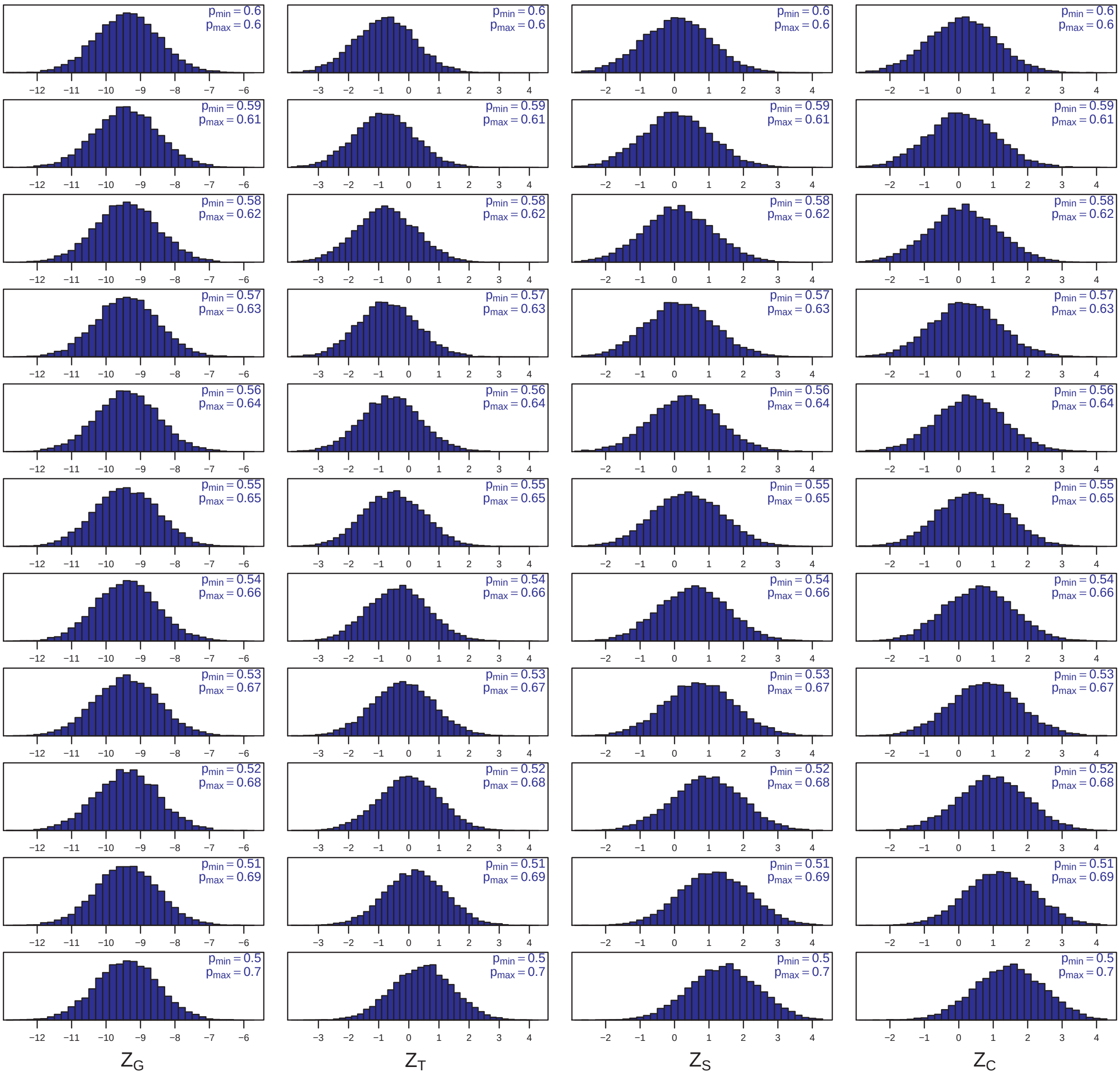

Figure 2. Method validation with simulations
Distributions of Z for different aggregation levels (games, tournaments, seasons and career: G/T/S/C) and different values of ΔP ($\Delta = 0$ in all plots). Different rows correspond to different values of ΔP, while different columns correspond to differetn aggregation levels and different values of ΔP's as indicated in the legends. One sees the shift in the distributions for accross the different rows for all aggregation levels but the games (G) as expected.

Table

References

1. Dorsey-Palmateer R, Smith G (2004) Bowlers' hot hands. The American Statistician 58: 38–45.
2. Martin DEK (2006) Hot-hand effects in sports and a recursive method of computing probabilities for streaks. Comput Oper Res 33: 1983–2001.

Ref. number	Player name	Number of games
13248	Chris Barnes	1501
11534	Walter Ray Williams Jr	1403
4646	Tommy Jones	1306
2122	Patrick Allen	1159
1978	Ryan Shafer	1137
7258	Mika Koivuniemi	1123
8642	Wes Malott	1113
9630	Pete Weber	1108
2213	Brad Angelo	1082
8678	Norm Duke	1017
2771	Parker Bohn III	991
1713	Michael Fagan	932
11093	Mike DeVaney	902
12233	Robert Smith	871
1119	Jason Couch	838
6779	Steve Jaros	835
3375	Michael Machuga	823
3741	Danny Wiseman	732
1991	Doug Kent	695
12759	Chris Loschetter	666
6076	Eugene McCune	662
20327	Sean Rash	640
2419	Joe Ciccone	613
8457	Mike Edwards	613
9680	Brian Voss	604
5457	Brian Kretzer	603
6077	Mike Wolfe	601
2333	Tommy Delutz Jr	561
11908	Amleto Monacelli	558
1544	Tom Baker	551
2021	Patrick Healey JR	542
11216	Michael Haugen Jr	535
10991	Tony Reyes	496
5913	Dave D'Entremont	492
6673	Jeff Carter	439
5543	Brian Himmler	437
3772	Tim Criss	416
9803	Lonnie Waliczek	404
9861	Rick Steelsmith	400
12410	Ronnie Russell	388
11228	Jason Hurd	385
35089	Rhino Page	362
19657	Dino Castillo	324
12479	Chris Johnson	320
8328	Paul Fleming	319
36058	Jason Belmonte	309
8721	Chris Warren	298
1919	Brian LeClair	297
8548	Rick Lawrence	288
9941	Hugh Miller	263

Table 1. Names of players included in the analysis along with their ref. number and number of games recorded

Ref. number	Player name	Number of games
30957	Billy Oatman	256
12476	Nathan Bohr	255
10645	Bryon Smith	249
11988	Steve Wilson	245
11460	Dennis Horan Jr	230
8015	Mitch Beasley	230
30015	Todd Book	229
3080	Bob Learn JR	220
4402	Christopher Collins	216
25886	Eddie VanDaniker Jr	215
12932	Troy Wollenbecker	213
33639	Ryan Ciminelli	205
3883	Richard Wolfe	197
5168	Jeff Lizzi	197
4581	Ken Simard	194
14831	Liz Johnson	191
12299	Eric Forkel	189
3208	Jim Tomek Jr	185
12283	Randy Pedersen	179
12108	Peter Hernandez	177
12444	Tom Smallwood	172
8707	D.J. Archer	168
4916	Tom Daugherty	167
13611	Kelly Kulick	159
121	Andres Gomez	156
7765	Riga Kalfas	154
4482	Randy Weiss	151
32863	John Nolen	138
8541	Del Ballard JR	134
8158	Stevie Weber	134
12483	Shannon Buchan	129
7516	Dale Traber	128
12623	Tore Torgersen	128
16774	Chad Kloss	127
12606	Tim Mack	127
6947	Jason Queen	126
25853	Jason Sterner	125
13610	Carolyn Dorin-Ballard	124
1574	Chris Hayden	120
11920	Steve Hoskins	113
6569	Mike Mineman	112
12173	Jim Pratt	105
12559	Patrick Girard	105
13964	Ken Abner	101
38705	Dan MacLelland	94
6429	Steve Rogers	93
34028	PJ Haggerty	90
9822	Bryan Goebel	90
2482	Rudy Kasimakis	88
13805	Bill Rowe	82

Table 1. Names of players included in the analysis along with their ref. number and number of games recorded
